# Complementary and Alternative Medicine in Cancer Stem Cells

**DOI:** 10.1155/2013/560274

**Published:** 2013-08-06

**Authors:** Hui-Fen Liao, K. S. Clifford Chao, Yu-Jen Chen, Min Shen Chang

**Affiliations:** ^1^Department of Biochemical Science and Technology, National Chiayi University, Chiayi 600, Taiwan; ^2^Department of Radiation Oncology, Columbia University, New York, NY 10032, USA; ^3^Department of Medical Research and Department of Radiation Oncology, Mackay Memorial Hospital, Taipei 104, Taiwan; ^4^Department of Ophthalmology and Visual Sciences, Vanderbilt University, Nashville, TN 37232, USA

Cancer stem cells (CSCs), a small subpopulation of cancer cells prone to form tumorigenesis, resemble normal stem cells with self-renewal and differentiation characteristics. CSCs have recently been identified in leukemia and various types of solid tumors. There is growing evidence indicating that CSCs might be the principal cause for the development of resistance to cancer treatments, recurrence, and metastasis. Emerging data indicates that CSCs and normal stem cells may share cellular pathways regulating proliferation and differentiation. These pathways include, but not limited to, polycomb group transcriptional repressor Bmi-1, Notch, Sonic hedgehog, and Wnt pathways. Therefore, identification of CSC markers, selection and development of unique therapeutics targeting CSCs and related signaling pathways have been extensively investigated. In this special issue, we publish articles that explore aspects of complementary and alternative medicine (CAM) associated with CSCs in basic research, in translational medicine, and in clinical application for cancer treatment. The topic will highlight current research, encourage additional research, draw attention to the subject, and ultimately help advance the field of CAM application in CSCs. 

Considering the widespread research on the role of CAM in CSCs, 1 review and 12 research articles have been included in the current special issue. Among the papers, Y. C. Huang et al. contributed with an excellent review about naturally occurring compounds and derivatives targeting Sonic hedgehog signaling. C. W. Chang et al. and Y. M. Liu et al. reported the effect of *Antrodia cinnamomea* against CSCs of head and neck cancer and hepatoma, respectively. Q. A. Jia et al. contributed a research on chemosensitization of hepatoma by using herbal compound “Songyou Yin.” Y. A. Shen et al. published data for resveratrol on stemness, epithelial-mesenchymal transition, and metabolic reprogramming of CSCs in nasopharyngeal carcinoma through p53 activation. Y. J. Chen et al. provided another bioactivity of resveratrol against alpha-melanocyte-stimulating hormone signaling, viability, and invasiveness in melanoma cells. Y. C. Su et al. further demonstrated the effect of resveratrol on downregulating interleukin 6-stimulated Sonic hedgehog signaling in human acute myeloid leukemia. W. Y. Liao et al. found that cyclohexylmethyl flavonoids could suppress propagation of breast CSCs via downregulation of NANOG. B. Yan et al. reported *β*-elemene as an antiangiogenesis agent targeting Notch-1 in gastric cancer stem-like cells. C. M. Lee et al. presented results of pterostilbene, a blue berry isolate, on suppressing irradiation-mediated enrichment of hepatoma CD133^+^ stem cells. For lung cancer, C. T. Yeh et al. demonstrated antimycin A as a potential anti-CSCs agent.

The aforementioned papers published in this special issue represent quite attractive and promising results about CAM in management of CSCs. In most of these papers, the mechanisms underlying their anti-CSCs properties have been well addressed.

